# Enhanced Humidity Sensing Response of SnO_2_/Silicon Nanopillar Array by UV Irradiation

**DOI:** 10.3390/s19092141

**Published:** 2019-05-09

**Authors:** Wei Li, Linlin Wang, Yun Cai, Peifeng Pan, Jinze Li, Qingying Ren, Jie Xu

**Affiliations:** 1 College of Electronic and Optical Engineering & College of Microelectronics, Nanjing University of Posts and Telecommunications, Nanjing 210023, China; 1217022824@njupt.edu.cn (L.W.); 1017020831@njupt.edu.cn (Y.C.); 1216022745@njupt.edu.cn (P.P.); lijinze@njupt.edu.cn (J.L.); Rqy@njupt.edu.cn (Q.R.); jiexu@njupt.edu.cn (J.X.); 2 School of Physics, Nanjing University, Nanjing 210093, China

**Keywords:** humidity sensing, SnO_2_ film, silicon nanopillar array, UV light

## Abstract

In this work, a silicon nanopillar array was created with nanosphere lithography. SnO_2_ film was deposited on this nanostructure by magnetron sputtering to form an SnO_2_/silicon nanopillar array sensor. The humidity sensitivity, response time, and recovery time were all measured at room temperature (25 °C) with UV or without UV irradiation. As a result, the humidity sensitivity properties were improved by enlarging the specific surface area with ordered nanopillars and irradiating with UV light. These results indicate that nanostructure sensors have potential applications in the field of sensors.

## 1. Introduction

Gas sensors with a metal oxide semiconductor (MOS) have received much attention in recent years [1,2,3,4,5,6,7,8]. MOS sensors depend on the change of a metal oxide semiconductor layer in resistance or capacitance, which is induced by the interaction between a surface and ambient gas. Many materials, such as ZnO, WO_3_, and SnO_2_, are designed as gas sensors [9,10,11,12,13,14,15,16]. Due to the large interaction energy of chemisorption, these MOS gas sensors have good performance at high temperatures to overcome the energy limitations of rapid response and recovery time. This places significant pressure on gas sensor reliability and durability at high temperatures. For this reason, many researchers have focused on the development of room temperature MOS sensors [17,18,19,20].

Tin oxide is a sensitive material with a band gap of Eg = 3.7 eV. Some recent papers demonstrated that nanostructured SnO_2_ as a gas sensor has considerable humidity sensitivity [21,22,23]. As mentioned above, the high operating temperature is still a significant issue. One possible solution is to enlarge the specific surface area [24,25]. With more surface area, more gas molecules are adsorbed. An alternative efficient and inexpensive method is to apply UV light irradiation [26,27,28].

In our previous papers, the preparation and characterization of a silicon nanopillar array sensor were reported [29,30]. In addition, enhanced humidity sensitivity of the silicon nanopillar array sensor by UV light was also achieved [31]. In this paper, SnO_2_ film was prepared by magnetron sputtering on a silicon nanopillar array to form an SnO_2_/silicon nanopillar array humidity sensor. With UV light irradiation, SnO_2_/silicon nanopillar array humidity sensing activity was improved. These results indicate that nanostructure sensors have potential applications in the field of sensors.

## 2. Experiment

### 2.1. Preparation of the Large-Area and Ordered Polystyrene Spheres Array

In this experiment, the Si nanopillar array was created by nanosphere lithography. A P-type silicon wafer (110) was used in this work. A long-range ordered polystyrene (PS) sphere monolayer was deposited on the silicon substrate by self-assembly. The PS spheres suspension (10 wt%) with 220 nm diameters was purchased from Duke Scientific Corporation (California, USA). An approximate 5 μL solution was applied to the silicon substrate by micropipette. The substrate with PS spheres was slowly dipped into deionized water in a glass basin, at which point the PS nanospheres slipped from the Si wafer to form a monolayer on the water surface. The monolayer was then taken to the prepared silicon wafer.

### 2.2. Preparation of Ordered Silicon Nanopillar Array

This silicon wafer was put into a reactive-ion etching (RIE) chamber. It was etched with CF_4_ (30 W RF, 20 Sccm) for 3 min. In this process, Si was etched with the following reactions:

CF_4_+e^−^ → CF_3_^+^ + F + 2e^−^ (1)

Si + 4F → SiF_4_↑ (2)

Here, the reducing rate of PS spheres is smaller than that of silicon. PS spheres were used as a mask and were reduced by plasma bombardment. As a result, the nanoscale pattern with PS sphere was transferred to form a silicon pillar array. In the next process, the substrate was put into a tetrahydrofuran (THF) solvent to remove all of the PS spheres.

### 2.3. Preparation of SnO_2_/Silicon Nanopillar Array Sensor

SnO_2_ film was deposited on the silicon nanopillar array by a sputtering instrument (JGP-560, SKY Technology Development, China) with the pressure of 1 × 10^−4^ Pa. A 99.99% pure SnO_2_ target was deposited on the above Si substrate under a current density of 60 mA/cm^2^, a power of 90 mW, and a deposition time of 2 min. After the sputtering process, the sample was thermally annealed at 1000 °C with oxygen for 1 h. The interdigital measurement electrodes of 1 × 1 cm^2^ were prepared on the SnO_2_ film surface. It was performed by electron-beam evaporation (EBV) in a vacuum of 3 × 10^−4^ Pa, with an evaporation current of 25 mA and a deposition time of 30 s. The width of the finger was 2 mm and the length was 10 mm. The gap parameters were the same as those of the finger. 

### 2.4. Measurement of Humidity Sensing Properties 

The humidity sensing properties of the SnO_2_/silicon nanopillar array were assessed by measuring its capacitance variation with relative humidity (RH) levels of 11%, 34%, 56%, 75%, and 90% at room temperature. These humidity environments were provided by containing salt solution (MgCl_2_, LiCl, KNO_3_, NaCl, KCl, and Mg(NO_3_)_2_,) in air–glass chambers. 

## 3. Discussion and Results 

**Figure 1:**
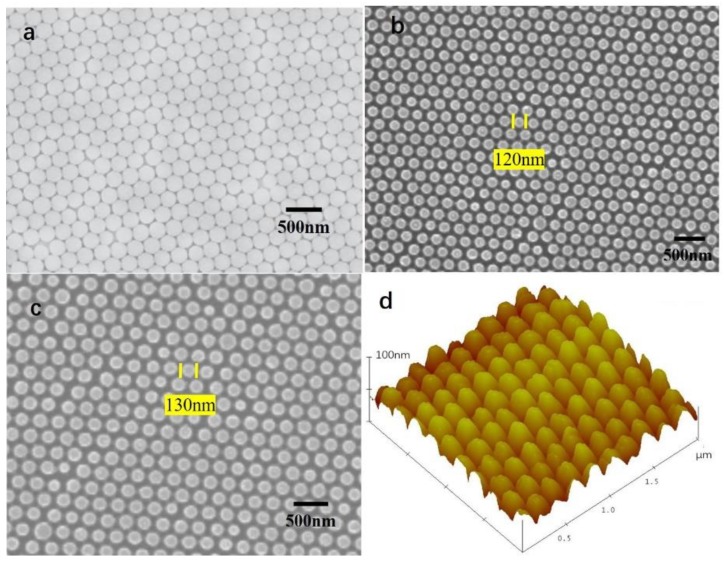
(**a**) Scanning electron microscopy (SEM) image of the ordered polystyrene (PS) sphere array. (**b**) SEM image of the silicon nanopillar array. (**c**) SEM image of the silicon nanopillar array with SnO_2_. (**d**) Atomic force microscopy (AFM) image of the silicon nanopillar array with SnO_2_.

**Figure 2:**
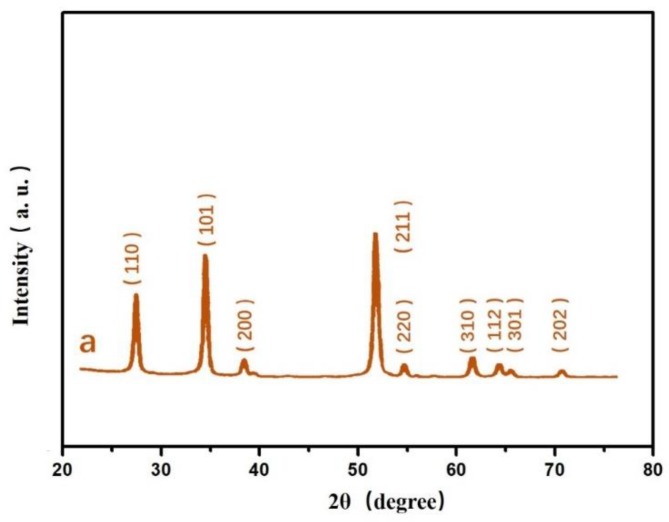
X-ray diffraction patterns of SnO_2_.

Figure 1a shows a scanning electron microscopy (SEM) image of the ordered PS sphere array. The periodic single layer PS sphere array was fabricated on a silicon wafer, which displayed a classic honeycomb, close-packed structure with a period of 220 nm (PS sphere diameter). Figure 1b shows an SEM image of the Si nanopillar array. The diameter of the pillar was approximately 120 nm. This demonstrates that the honeycomb, close-packed structure was well transferred from the PS sphere to the silicon wafer surface. As shown in Figure 1c, the SnO_2_ film was deposited onto the silicon nanopillar array. The surface morphology of the silicon nanopillar array with SnO_2_ was maintained without decomposition. The size was 130 nm, which was bigger than the fresh silicon nanopillar array. Figure 1d is an atomic force microscopy (AFM) image of the silicon nanopillar array with SnO_2_ film. It demonstrates the oblique view of the silicon nanopillar array with SnO_2_ film. The height of pillar was approximately 110 nm. The X-ray diffraction patterns of the SnO_2_ film are shown in Figure 2. These indicate that a SnO_2_ film with a crystalline property was homogeneously deposited on the Si nanopillar array.

**Figure 3:**
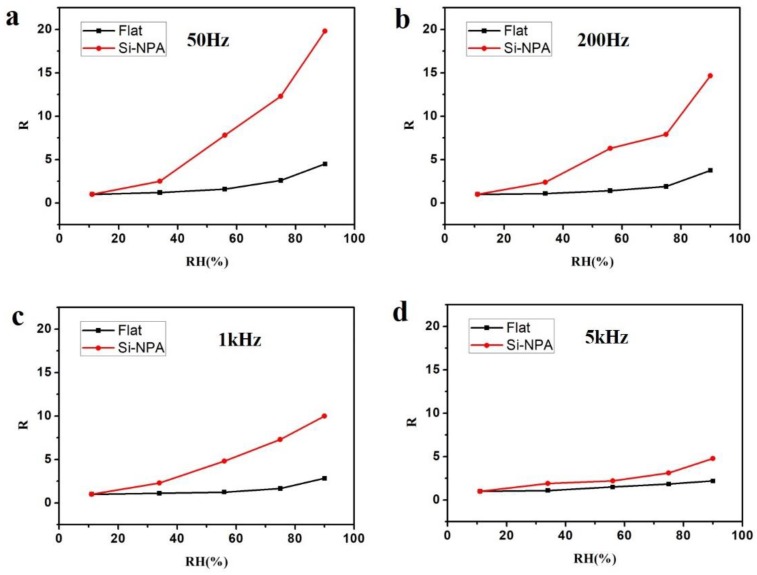
Humidity response curves measured under four frequencies: (**a**) 50 Hz, (**b**) 200 Hz, (**c**) 1 kHz, (**d**) 5 kHz.

In our case, the capacitance response was measured under four different frequencies—50 Hz, 200 Hz, 1 kHz, and 5 kHz. The SnO_2_/silicon nanopillar array humidity sensing activity was studied with UV light irradiation. As a point of comparison, the SnO_2_ film on the flat surface silicon wafer (SnO_2_/Si) was also studied. Here, the humidity response R is defined as:

(3) $$R=\frac{C_{RH}}{C_{11}}$$

where *C*_11_ is the capacitance at RH = 11% and *C*_RH_ is the capacitance at a certain RH (34%, 56%, 75%, and 90%). Figure 3 shows the humidity response curves measured under four frequencies. As seen in Figure 3, all four figures show the same result, that humidity response for SnO_2_/silicon nanopillar array sensors increased with RH increasing, which indicates that SnO_2_/silicon nanopillar arrays can be used as humidity sensors. The response of the SnO_2_/silicon nanopillar arrays was better than that of SnO_2_/Si, as shown in Figure 3. This phenomenon might have been caused by enlarging the specific surface area with an ordered nanopillar array. The more specific surface area, the more water vapor was adsorbed. Furthermore, humidity responses decreased with the increase in applied frequency. It is because of this that water molecules can be polarized in an alternating electric field. Past studies have reported that polar water molecules are able to follow an alternating electric field with a relatively short relaxing time at low frequency, but a long relaxing time at a high frequency [32,33]. Capacitance is proportional to dielectric constant. When the testing frequency was 50 Hz (low frequency), the speed of the alternating electric field was low and the polarized water molecules were likely to follow. At this frequency, the dielectric constant remained stable. When the testing was 200 Hz, 1 kHz, and 5 kHz (high frequency), the speed of the alternating electric field was high and it was difficult for polarized water molecules to catch up. This results in the decrease of the dielectric constant, which causes an abatement of the sensor capacitance. 

**Figure 4:**
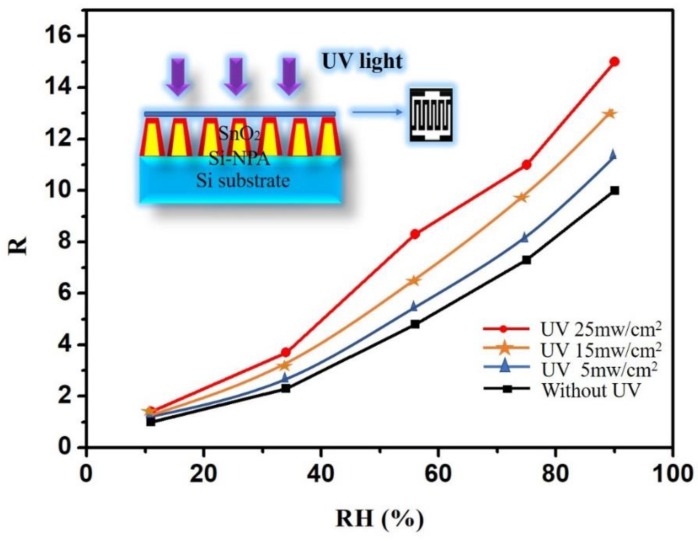
The humidity responses with and without UV. The inset illustrates the schematic diagram of the SnO_2_/silicon nanopillar array sensor with UV light.

To improve humidity sensitivity, UV-activated irradiation was carried out. These measurements were studied in a quartz glass under UV light irradiation at room temperature (25 °C). UV light was provided by a light-emitting diode (LED). Considering both the linearity and the response of the abovementioned studies, the applied frequency was 1 kHz. The humidity responses with and without UV are shown in Figure 4. The inset illustrates the schematic diagram of the SnO_2_/silicon nanopillar array sensor with UV light. Three different power intensities (25 mW/cm^2^, 15 mW/cm^2^, and 5 mW/cm^2^) of UV light with a wavelength of 300 nm were applied in our work. As shown, increasing power produced a greater response. The optimal response was obtained with 25 mW/cm^2^. The response increased across the entire testing RH range with UV light. Table 1 shows the outcome of the humidity response with UV (25 mW/cm^2^) and without UV. As can be seen from Table 1, the increase was 20% at 11% RH. In the other case, the increase was approximately 50%. The maximum response increase was 58% at 56% RH. 

**Figure 5:**
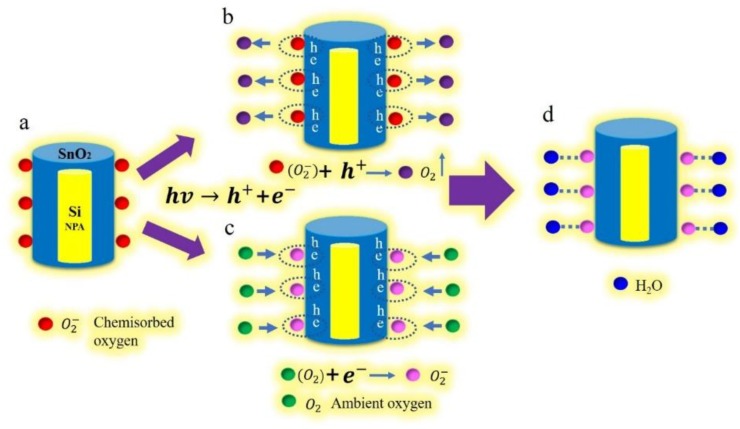
The UV-activated humidity sensing mechanism of the silicon nanopillar array sensor.

The humidity sensing mechanism of the UV-activated silicon nanopillar array sensor is illustrated in Figure 5. The photogenerated electrons and holes, induced by UV light, might be a major contributor. Without UV irradiation, the chemisorbed oxygen ion (O_2_^−^) is thermally stable. Due to the large adsorption energy, it is difficult to be removed from the SnO_2_ surface (Figure 5a) [34]. With UV irradiation, the electrons and holes are generated on the interface between SnO_2_ and the silicon nanopillar array. The adsorbed oxygen ions interact with the photoinduced hole, causing the oxygen gas to be desorbed according to the following reaction (Figure 5b):

(4) $$hv\to h^{+}+e^{-}$$

(5) $$O_{2}^{-}+h^{+}\to O_{2}↑.$$

Meanwhile, the ambient oxygen molecules interact with photoelectrons to form the additional photoinduced oxygen ions as shown in the following scheme (Figure 5c):

(6) $$O_{2}+e^{-}\to O_{2}^{-}.$$

**Figure 6:**
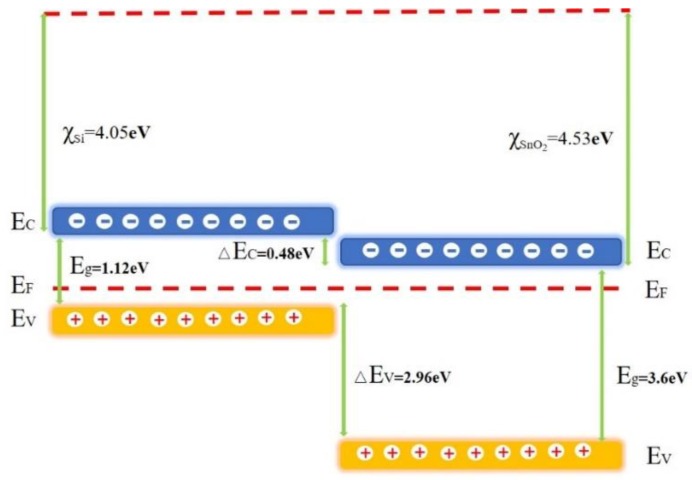
The energy band diagram of the nanostructure of SnO_2_/Si.

These additional photoinduced oxygen ions are combined with water molecules by covalent bonds (Figure 5d). Figure 6 shows the energy band diagram of the nanostructure of SnO_2_/Si according to the Anderson model. E_F_ is the Fermi level position, Ec and Ev are the conduction and valence band edge, Eg is the band gap, △E_C_ and △E_F_ are the band discontinuities, χ_Si_ and χ_SnO2_ are the electron affinity energies of Si and SnO_2_. Under UV irradiation, SnO_2_ and Si absorb photon energy to generate electrons and holes. As χ_Si_ is smaller than χ_SnO2_, the electrons flow from the Si conduction band to SnO_2_. The process was enhanced as more electrons appeared on the surface of SnO_2_. Therefore, more water molecules were absorbed with UV irradiation to condense liquid water than without UV. It was found that the sensor capacitance raises rapidly to improve the humidity sensitivity.

**Figure 7:**
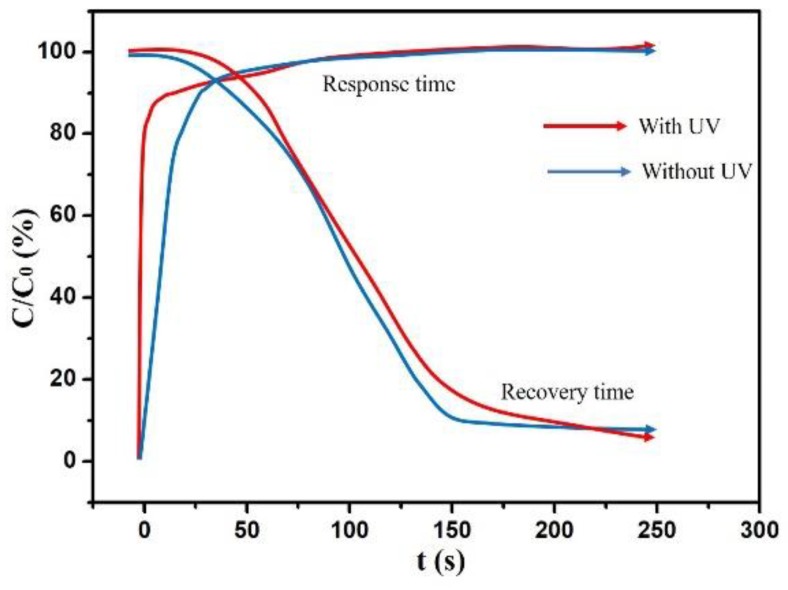
The response and recovery times under RH = 90%.

**Figure 8:**
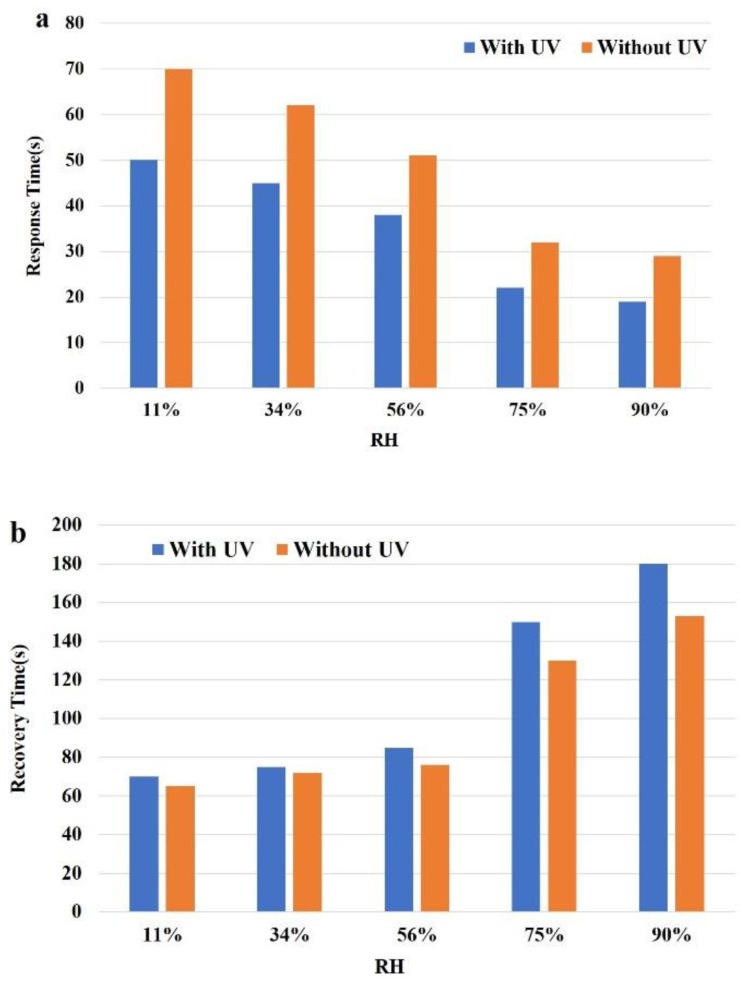
(**a**) The response time of the SnO_2_/silicon nanopillar array sensor. (**b**) The recovery time of the SnO_2_/silicon nanopillar array sensor.

The response and recovery time of the sensor was also measured. C is the instantaneous capacitance of this SnO_2_/silicon nanopillar array sensor, and C_0_ is either the final or initial value. The response time is defined as the time taken to go from C/C_0_ = 0% to 90%. The recovery time is defined as the time taken to go from C/C_0_ = 100% to 10%. Here, the response and recovery times were measured by switching the humidity between RH = 11% and 90% under the frequency of 1 kHz. Figure 7 shows the response and recovery time under RH = 90%. With UV, the response time was 19 s and the recovery time was 180 s. Without UV, the response time was 28 s and the recovery time was 155 s. Furthermore, Figure 8a and Figure 8b show the response and recovery times when the relative humidity switches from 11% RH to 34%, 56%, 75%, and 90%, respectively. Both with and without UV irradiation, the response time decreased with the increase of humidity. However, the recovery time increased with the increase of humidity under UV irradiation. This is mainly because more water vapor condenses liquid water on the surface of SnO_2_/silicon nanopillar array at the same time under a high RH, but the liquid water becomes more difficult to desorb with the increase of humidity. The fastest response time was 19 s at 90% RH with UV and the fastest recovery time was 65 s at 11% RH without UV. Table 2 summarizes the humidity sensing performances of our work and other humidity sensors reported. It notes that the performances of our sensors are significantly superior to others, not only in terms of sensitivity but also in response and recovery times.

## 4. Conclusions

In this study, SnO_2_/silicon nanopillar array sensors were prepared. With UV light irradiation, SnO_2_/silicon nanopillar array humidity sensing activity was improved. As a result, the humidity sensitivity response showed better linearity at the testing frequency of 1 kHz. Furthermore, the increase in response reached above 50% with UV irradiation. With the increase of humidity, the response time decreased and the recovery time increased. The fastest response time was 19 s and the lowest recovery time was 180 s at 90% RH with UV. The lowest time was 50 s and the fastest recovery time was 65 s at 11% RH without UV. 

## Figures and Tables

**Table 1:** The humidity responses with and without UV.

	11% RH	34% RH	56% RH	75% RH	90% RH
Without UV Response	1	2.3	4.8	7.3	10.1
With UV Response	1.2	3.5	8.6	10.8	15.4
Response Increase	20%	52%	58%	48%	52%

**Table 2:** The performances of humidity sensors from this work and other reports.

Materials	RH	Sensitivity	Response Time (s)	Recovery Time (s)	Reference
SnO_2_/silicon nanopillar array	90%	16	19	180	This work
SnO_2_	95%	~2	26	45	[35]
SnWO_4_-SnO_2_	90%	~4	30	100	[36]
MoS_2_/Si	90%	3	36.3	57.6	[37]
Graphene	84%	2	180	180	[38]
